# Parents’ and guardians’ perceptions on availability and pricing of medicines and healthcare for children in eThekwini, South Africa – a qualitative study

**DOI:** 10.1186/s12913-017-2385-y

**Published:** 2017-06-19

**Authors:** Velisha Ann Perumal-Pillay, Fatima Suleman

**Affiliations:** Discipline of Pharmaceutical Sciences, School of Health Sciences, Westville Campus, University of KwaZulu-Natal, Private Bag X54001, Durban, 4000 South Africa

**Keywords:** Access, Availability, Affordability, Essential medicines, Private sector healthcare, Public sector healthcare, South Africa

## Abstract

**Background:**

Inadequate access to affordable essential medicines poses a challenge to achieving Universal Health Coverage. Access to essential medicines for children has been in the spotlight in recent research. However, information from the end users of medicines, i.e. patients is scarce. Obtaining information at a household level is integral to understanding how people access, obtain and use medicines. This study aimed to gather opinions and perceptions from parents/guardians on availability, affordability and quality of medicines and healthcare for children in SA.

**Methods:**

Eight Focus group discussions were held with 41 individuals in eThekwini, South Africa (SA), from September–November 2016. Participants were parents/guardians of children up to 12 years from different ethnicities, ages, gender, and socio-economic backgrounds. Key informants identified by the principal researcher recruited participants using snowball sampling. Focus group discussions were recorded, transcribed verbatim, coded by the first author, verified by the second author, reconciled for consensus and imported into NVIVO for data analysis.

**Results:**

Medicines and healthcare facilities are accessible in urban and peri-urban areas in eThekwini. Medicines may not always be available in public sector facilities due to medicine shortages, compelling parents to purchase medicines from private sector pharmacies. Common medicines were perceived as affordable for most socio-economic groups except the ‘Poor’ group. Quality of medicines was perceived as ‘good’ especially if obtained from the private sector but sometimes perceived as ‘poor’ and viewed with suspicion when received from public sector clinics. Quality of healthcare was perceived as ‘good’ but requires improvement for both sectors.

**Conclusions:**

This is the first study in SA to report on parent/guardian perceptions on availability, affordability and quality of medicines and healthcare for children. It has the potential to be up-scaled to a country-wide investigation to paint a national picture of parents’ opinions of healthcare for children. This will allow for patient input into pharmaceutical and healthcare policy governing access to and availability of essential medicines and services within the country. The study recommends that patient input be sought to assess impact of policies on the intended target group in the country to ensure that the policy objectives are achieved.

**Electronic supplementary material:**

The online version of this article (doi:10.1186/s12913-017-2385-y) contains supplementary material, which is available to authorized users.

## Background

Globally, a quarter of all health expenses is on medicines [1]. Medicines account for 20–60% of health expenditure in developing countries [2]. In many countries, the primary source of funding medicines is through out-of-pocket payments by individuals and households [1]. This method of sourcing medicines is both inequitable and inefficient and poses a challenge to the concept of universal health coverage which aims to ensure all individuals and communities receive the required health services without experiencing financial difficulties [3]. Universal health coverage includes the full spectrum of essential, quality health services, from health promotion to prevention, treatment, rehabilitation, and palliative care [3]. Access to affordable essential medicines is important in this respect.

Access to essential medicines is a fundamental human right. The “right to health” is enshrined in international law. The concept first emerged as a social right in the World Health Organization (WHO) Constitution in 1946 [4], then in the Universal Declaration of Human Rights in 1948 [5]. The International Covenant on Economic, Social, and Cultural Rights of 1966 also describes the right to health through four steps, including access to health facilities, goods and services [6].

Examples of initiatives to enhance access to affordable medicines include the following: (a) the tendering processes by preference policy in The Netherlands, where medicines are procured within certain classes and the manufacturer with the lowest price is awarded the tender [7]; (b) the Wise List in Stockholm, Sweden, which contains approximately 200 medicines catering for more than 90% of the needs in ambulatory care with over 90% adherence rates. Costs are reduced by the availability of low prices for generics and mandatory generic substitution [8]; and (c) the Novartis Access programme in Kenya aims to provide treatment for non-communicable diseases with 15 affordable medicines at a cost of USD 1 per treatment per month [9]. Thus, the availability of affordable generics and the implementation of measures to enhance their uptake should ultimately enhance universal access.

Essential medicines are medicines that satisfy the priority health care needs of the population [10]. Particular attention has been placed on essential medicines for children in recent years and in 2007 the WHO released an essential medicines list particularly for children which is currently in its 5th edition [11]. In addition, the WHO has also published a list of priority life-saving medicines for women and children in 2011 (updated in 2012) [12] and is also responsible for the “make medicines child size” campaign, which promotes the accessibility of safe, effective and quality medicines for children [13]. These lists and initiatives are meant as tools for countries to develop their own national essential medicines lists to meet the healthcare demands of specific populations and to escalate the accessibility of essential medicines for children. Despite the right to health, access to essential medicines, especially for children, remains a challenge in developing countries [14].

The move to a single health system, in South Africa (SA), in the form of National Health Insurance financing supports the idea of universal health coverage and is a step towards attaining South African citizens’ ‘right to health’ (and therefore medicines) as set out in the South African constitution [15]. SA has a national medicines policy and national essential medicines list since 1996 [16]. The new democratic government also instituted a number of policies and interventions to promote increased access to healthcare and essential medicines over the years. These included: (a) free healthcare to mothers and children up to the age of 6 years in 1994, including vaccines for children; (b) mandatory generic substitution in 2003 and other amendments to the Medicines and Related Substances Act of 1965 (c) to allow parallel importation of medicines, (d) the establishment of a medicines pricing committee (e) the introduction of a transparent pricing system for medicines and single exit price of medicines; (f) as well as the prohibition of bonusing and sampling practices in the sale of medicines in 1997 (these medicine pricing regulations were introduced in 2004); (g) amendments to the Pharmacy Act (Act 53 of 1974) in 1997 which extended ownership of pharmacies to non-pharmacists with the intention of increasing access to pharmacy services especially in rural areas; and (h) the regulation of prescribed minimum benefits in the Medical Schemes Act (Act131 of 1998); (i) the implementation of a central chronic medicine dispensing and distribution programme (CCMDD) in 2014 at 10 National Health Insurance districts, is intended to improve access to chronic medicines, service delivery and enhance patient experiences by reducing waiting times and allowing medicines to be collected from alternative pick-up points, including private sector pharmacies [17]. Despite these measures access to medicines is still problematic in the country [18]. The provision of healthcare in SA is through both public and private sectors. Care at a Primary healthcare level in the public sector is free, with small fees at the higher levels. Healthcare in the private sector is primarily through medical insurance cover [18].

There are currently indicators and tools to measure access to medicines for which data is collected at a health care facility or pharmacy level [19]. However, not much information is available from the end users of medicines. The indicators measured at a health care facility/pharmacy level are useful but obtaining information at a household level is vital to obtain accurate information on how people access, obtain and use medicines [20]. This study therefore aimed to gather information (opinions and perceptions) from parents and/or guardians, during focus group discussions, on availability and pricing of medicines and healthcare for children. The study setting was eThekwini region in SA. To the best of our knowledge, a study of this nature reporting qualitative responses on medicines and healthcare availability and pricing for children, from parents/guardians has not been done in SA.

## Methods

### Sample selection, response rates and description of the sample

The study was conducted in eThekwini, Durban, South Africa from September – November 2016. A total of 8 focus group discussions were held with groups ranging from 4 to 6 individuals. These individuals were recruited from various areas in eThekwini to geographically represent the North, South, and Central regions. These locations included urban and peri-urban areas including 3 townships as well. These locations for sample recruitment were purposively selected with the hope that a range of individuals across racial and socio-economic lines would be included in the sample. Of the 44 individuals recruited, only 41 agreed to participate. An important inclusion criteria was that participants had to be parents and/or guardians of one or more children aged 12 and under.

Participants were recruited through a two-step process whereby 8 key informants were identified by the principal researcher; thereafter these key informants recruited the remaining participants using snowball sampling technique [21]. The characteristics of the participants are described in Table 1. All participants were fluent in English; however a Zulu translator was present if needed. The researchers intended to include individuals from different socio-economic groups, thus key informants from both urban and peri-urban areas in eThekwini were selected. The key informants were known to the principle researcher through daily interactions over the years. These key informants went back to their places of residence where further recruitment took place. This technique of recruitment was particularly useful for recruiting participants in areas unfamiliar to the researchers as it is unsafe to travel alone into unfamiliar areas and townships due to high crime in SA [22].

**Table 1:** Characteristics of the study sample

Focus Group (number of Participants)	Age Range (years)	Gender	Ethnicity	Education	Employment	Income Group	Medical Insurance
1 (6)	21–28	Female	African	Secondary Tertiary	Teaching assistants	Poor	No
2 (5)	24–34	Female(4) Male(1)	African	Primary Secondary	Garden service	Poor	No
3 (6)	22–46	Female	African	Secondary Tertiary	Unemployed(4); Admin Clerk; Cleaner	Low Emerging Middle Class	Yes(1)
4 (6)	28–42	Female(3) Male(3)	Indian(5) African(1)	Secondary Tertiary	Driver; Admin clerk; Pharmacist assistant; Professional nurse; Nanny; Security guard	Low Emerging Middle Class	Yes(2)
5 (5)	22–55	Female	Indian(2) Coloured(3)	No Formal Secondary Tertiary	Admin (2); housewife (2); Team Leader	Realised Middle Class	Yes(3)
6 (4)	29–40	Female	Indian(2) African(2)	Tertiary	Self-employed; locum pharmacist; Educator; housewife	Realised Middle Class	Yes
7 (4)	32–39	Male	Indian	Secondary Tertiary	Director: Civil construction; Logistics Manager; Financial Advisor; Regional Manager cell company	Upper Middle Class	Yes
8 (5)	39–49	Female	White	Tertiary	Photographer(2); housewife; Décor coordinator; Estate agent	Emerging Affluent	Yes

Although SA is in a democracy since 1994, the racial and socio-economic segregation still exists and most people still reside in the previous apartheid system classified racial areas. It was possible to recruit individuals from all race groups (Africa, Indian, Coloured, and White) from most socio-economic backgrounds. A household income by income group for 2011, published by the Bureau of Market Research, University of South Africa was used to classify the socio-economic status of the groups, in terms of income per annum, as follows^1^ [23]:

- Poor (R0 - R54 344 (0–4170 USD)
- Low emerging middle class (R54 345-R151 727 (4171–11,553 USD))
- Emerging middle class (R151 728-R363 930 (11554–27,712 USD))
- Realised middle class (R363 931-R631 120 (27713–48,120 USD))
- Upper middle class (R631 121-R863 906 (48121–66,484 USD))
- Emerging affluent (R863 907-R1 329,844 (66485–101,396 USD))
- Affluent (R1 329,845+ (101,397+ USD))

Each group was classified according to the group average of income per annum per household. The sample included participants who were end users of both the public and private sectors of healthcare, with 19 (46%) participants being members of a private medical insurance scheme. Thus, we were able to capture responses and opinions on medicines and healthcare for both sectors of healthcare. The study sample is described in more detail in Table 1 below.

Focus group discussions are a useful qualitative method to gather opinions, observations, experiences and perceptions from individuals of a similar background. It provides insight into how a particular group thinks and can be used to explore meanings of survey findings that cannot otherwise be explained with statistics. It is also useful in providing insight into different opinions amongst different groups and plays a role in bridging research and policy involved in the change process. It therefore enables more efficient management of the process [24].

### Development of the instrument

The questions for the focus group discussions were derived from the standard WHO access to and use of medicines survey [25]. The first draft of the discussion guide was piloted with a parent of 2 children up to the age of 12 years. The discussion guide was then amended as recommended during this piloting process to ensure questions were clear and not ambiguous or confusing for parents. The discussion guide (see Additional file 1: discussion guide) comprised questions for demographic and socio-economic information as well as open ended questions concerning medicines and healthcare for children. The discussion guide sought to answer the following questions [26]:

Do people have access to medicines and healthcare; are medicines available in the public sector; are medicines and healthcare geographically accessible in the urban and peri-urban areas; are medicines for common conditions (both acute and chronic) affordable, especially for low-socioeconomic groups; how widespread is medical insurance coverage; who prescribes medicines and where do households purchase medicines?

### Data collection, processing and analysis

Eight focus group discussions facilitated by the first author were conducted in English, and audio-tape recorded. Discussions were held in easily accessible neutral venues at a date and time convenient to all participants and lasted between 40 and 60 min.

The audio-tape recordings were transcribed verbatim. Transcripts were verified and subsequently coded with the aid of NVIVO (version 10) software for qualitative data analysis. A thematic analysis of the data was performed based on grounded theory. Data was coded and classified and then re-classified into sub-codes to ensure no new themes emerged from the data. The data and codes were reviewed and discussed with the second author for consensus on coding and emergent themes.

### Ethics statement

The study was granted ethical clearance by the University of KwaZulu-Natal Human and Social Sciences Research Ethics Committee (HSS/0154/013). Prior to participation, parents and guardians were briefed on the study objectives, methods of data collection and consent was sought to publish the findings as aggregated responses to maintain confidentiality. Recruited individuals who agreed to participate signed informed consent forms prior to the focus group discussions.

## Results

The key themes that emerged from the focus group discussions were: 1. Access to and availability of healthcare services and medicines for children; 2. Affordability of medicines and healthcare services for children; 3. Quality of medicines and healthcare services for children. These are discussed separately in the sections to follow.

### Access to and availability of healthcare services and medicines for children

It was found that all participants were able to access healthcare and medicines for their children at either public sector primary healthcare clinics and/or private sector General practitioners, clinics and hospitals. The 2 ‘Poor’ income groups used only the public sector for all healthcare services but were sometimes forced to use private sector pharmacies due to shortages or unavailability of medicines at the public sector clinics. Participants from all other groups used a combination of health sectors and it was found that at least one participant in each of the remaining socio-economic groups used the public sector clinics for immunizations. The main reason for this was because it was a free service whilst those who preferred to use the private sector had to pay for this service. The latter was an option for those who belonged to a medical scheme and participants either paid in full or just a co-payment depending on their benefit option with the medical scheme.

Access to healthcare in terms of travelling time to the nearest healthcare facility varied between groups. Some participants from the ‘Poor’ and ‘Low emerging middle class’ reported that it could take approximately 1 h to travel to the facility (participants sometimes walked or waited that long for public transport). The remaining groups reported a maximum of 30 mins travelling time implying facilities were easily accessible and transport was not an issue.

Although some users of the public sector clinics said medicines were always available (‘Low emerging middle class’ group), the majority of users (‘Poor’ groups) complained that availability of medicines and vaccines was problematic. When participants were asked to comment on the availability of medicines at the facility they visited, their comments included:

“Sometimes they say they haven’t got, they running short, they say I must bring the child back. Like last month, I was going to the immunization; he was getting immunization for 18 months. They gave me only one, one injection. They said there is no other injection I must come back, and they give me”. (‘Poor’ group)

“I also go to the pharmacy sometimes because they complain about out of stock, and sometimes they don’t have medicine at the moment”. (‘Poor’ group)

“They don’t always give medicines at the clinic, and if the child has flu they tell you must use honey and lemon, they not giving medication, so it’s better to go in the pharmacy”. (‘Poor’ group)

Another participant from the ‘Low emerging middle class’ responded as follows:

“With regards to the clinic. They wouldn’t have put something there on the list or whatever they require if they don’t have it. They don’t ask you to buy anything privately…basically, he’ll give you a prescription, you go to their dispensary, collect whatever they have and if they don’t have, they give you something else which is in their dispensary to do the same thing”.(‘Low emerging middle class’ group)

By direct contrast, access to healthcare and availability of medicines in the private sector was not an issue. Some participants’ comments are provided below:

“I also don’t have a problem with medication. Fortunately I am able to have a medical aid plan that allows me to take my children and readily get whatever’s available and I think every mother at home always has Panado syrup and the basics of whatever medication we need in case our children get sick. So I don’t have any problems with medication or access to it”. (‘Low emerging middle class’ group)

“But generally the pharmacy we go to has everything, very rare you looking for something, so even if they don’t have they will order it”. (‘Upper middle class’ group)

“Always available, even if not at a pharmacy, maybe even at a store for example, Checkers and wherever”. (‘Low emerging middle class’ group)

### Affordability of medicines and healthcare services for children

It was evident from the interviews that participants from both sectors of healthcare visited facilities for both acute and chronic illnesses. Spending to treat these illness varied greatly per group with ‘Poor” groups spending up to R6000 (456 USD) per annum and up to R20 000 (1537 USD) for the ‘emerging affluent’. The difference could be attributed to the type of medicines purchased for the differing medical conditions as well as government subsidised healthcare in the public sector.

The most common conditions for which over-the-counter medicines were purchased were the common cold and flu; diarrhoea; pain and fever; and allergies. The most common medicines purchased were Panado® (paracetamol); Allergex® (chlorphenamine); cough syrups; antiseptic and antibiotic creams; and multivitamins. Participants most often spent on average approximately R2500 per annum and one participant spent up to R8000 per annum on over-the-counter medicines.

Irrespective of the socio-economic level, all participants were of the opinion that medicines were indeed expensive, although considered affordable by the middle class and affluent groups. Participants commented that some medicines such as antibiotics; nasal sprays; accuhalers for asthma, EpiPen® for peanut allergy; and ophthalmic drops were expensive. Participants also mentioned that generics were much cheaper than branded medicines. Some comments on the affordability of medicines are included below:

“Not affordable, over R1000pm for Ritalin®”. (‘Emerging affluent’ group)

“I find them affordable. My pharmacist will tell me if the doctor’s prescribed something expensive and suggest an alternative”. (‘Emerging affluent’ group)

“I think it’s sometimes expensive, because I have 3 kids. I like every month get them vitamins, I like to get them Scotts® and all that and the stuff and the syrups that the child needs but they so expensive I cannot afford them”. (‘Poor’ group)

“Whenever I buy something, I always think of people who can barely afford it, how do they afford it, how are they able to get the antibiotic at that price R300, R400…everyone wants their child to be better. So I always put myself in that situation. So in that respect, I think it’s very pricey for people who can’t afford it”. (‘Realised middle class’ group)

“Why is medicine overpriced because it’s not a luxury, it’s a necessity for people. So why is it so overpriced?” (‘Realised middle class’ group)

Comments with respect to affordability of healthcare services in the private sector highlighted discrepancies in pricing of services:

“If you look at a doctor’s consult and dispensary, the medication you get from the pharmacy is pretty much the same. It’ll cost you nothing less than a R1000 on a GP, than a pharmacy”. (‘Upper middle class’ group)

“I feel that the prices are extremely high and on medical aid it’s, I think it’s even worse…The prices on medical aid, I feel, is over-priced but at the same time it’s available to me…I am paying medical aid so my medical aid subsidies whatever I need. So I’m not really stressed about my spending out of my own pocket. But I do agree that it is expensive” (‘Low emerging middle class’ group)

“My issue is with the medical aid, with the pricing between the pharmacies and the doctors. I feel it’s exorbitant and it sometimes it’s like, instead of getting the proper medication, you are offered the generic because of the pricing and some of the medical aids don’t pay for the proper medication and well my issue is with that, whether the generic is going to do the same effect as the proper medication”. (‘Low emerging middle class’ group)

“The only thing I don’t understand as well, if you look at dispensing doctors, a dispensing doctor charges you a fee, say R300, they give you everything. You go to a non-dispensing doctor, he’ll charge you R300 plus you have to go spend another R700 for medication. How do they justify the pricing?” (‘Upper middle class’ group)

“It’s very pricey, especially when you get admitted. If you in hospital for about a week, that’s a chunk of your medical aid funds that’s gone and you actually worry that your child doesn’t get admitted again in the year and sometimes you have to pay more co-payments because not all the doctors charge 100%. The doctors are not charging 100% anymore, they’re charging 300% and your medical aid only covers 100%”. (‘Realised middle class’ group)

### Quality of medicines and healthcare services for children

When participants were asked their opinion on the quality of medicines, most responded in terms of the medicine ‘working’ or ‘not working’ for their child. Whilst many agreed medicines were of ‘good’ or ‘excellent’ quality, some mentioned that there was nothing to compare with and medicines were ‘average’ and participants basically assumed the medicines did was it was intended to do. Some said medicines do not work fast enough. The views of participants from the ‘Poor’ group was a cause for concern as these participants eluded to suspicions of medicines being tampered with at the public sector clinics they used. These participants indicated that this affected the quality of some medicines and resulted in the medicine not always working and these participants prefer medicines from the private sector pharmacies.

When participants were asked their opinion on the quality of healthcare services they received, responses were varied between public and private sectors users. Those that used the public sector felt that the availability of services were good despite the long waiting times but marred by the lack of stock of medication and negative attitude of the nurses who attended to them. The opinions for the private sector were a direct contrast where most participants felt that the service although expensive was excellent. However, a few related negative experiences.

A common problem experienced by both sectors was that of relationships with the healthcare professionals. In the public sector respondents felt the nurses were ‘rude’ and ‘shouting’ at them. Participants felt disrespected. The private sector users felt that they were not afforded enough time and information from their respective doctors for the amount of money they were spending to access these professionals. Some notable comments on the quality of medicines and healthcare services are described in Table 2.

**Table 2:** Participants’ perceptions on quality of medicines and healthcare services

	Public sector	Private sector
Quality of medicines	“I think this medication that we purchase over the counter that we pay for in the pharmacy is really good. It helps even if it’s very expensive for us. It’s good compared to the clinic, it’s really good. Even the clinic does have Panado, I know they always have Panado® and Allergex® but sometimes they add water and sometimes it’s not strong. Sometimes it’s expired and we can’t go to the clinic and return it because it’s expired, but over the counter at the pharmacy I can return it and say it’s expired. So I think the pharmacy is very good”. (‘Poor’ group) “The medication from the private pharmacy is really good. It helps. You can see it all the time, even the expiry date, you can check the expiry date. Sometimes in the clinic you don’t even see the expiry date. Sometimes the Panado is open, they give you with water and it is weak. So you have to go to the pharmacy. The pharmacy medication is really good.” (‘Poor’ group) “The ones that I’m buying I think they are very good. The quality is perfect because first the medication is served and it really helps. If you buy Panado®, you give the child, you monitor the child, the temperature will go down. So the medicine is very, very good. It is better than what you get from the clinic because sometimes they add lots of water. The medication that I buy from pharmacy is 100% good than the medication I get from the clinic.” (‘Poor’ group)	“What am I going to judge the quality against? It’s hard to judge because we only know the medicines here, we can’t compare it to anything, but generally I think it sorts the problem out”.(‘Upper middle class’ group) “Way better, my brother from London, whenever he comes down he takes a travel kit back up, so our medication is pretty good. The quality is good”. (Upper middle class’ group) “Considering my kids, the medicines do work because I have one that’s chronic, asthmatic, so I can’t say that the medication doesn’t work, it does”. (‘Realised middle class’ group) “Sometimes I have to go back to my pead (paediatrician) because it’s not working…average”. (‘Realised middle class’ group) “I think the generic is better than the original, generic is sometimes, when it comes to her, it works better”. (‘Realised middle class’ group)
Quality of services	“the quality of the health care I can say for the public clinic it’s not very bad except for the fact that they don’t have medication and stuff, because you don’t leave the clinic without your child being examined, they tell you exactly what is wrong with your child and they give a prescription if they don’t have that medication, but they don’t let your child just go out without trying to help him. Since it’s the government, I think they really try, it’s not very bad”. (‘Poor’ group) “The quality of the clinic is really, really good, we go there sometimes, but the only reason we end up buying it is because they out of stock”. (‘Poor’ group) “The care is excellent between both public and local GP’s… I should say public is a bit slow but we still receive the care, the best of care. It is time consuming to go publicly but we still receive excellent care”. (‘Low emerging middle class’ group) “The resources in public are very bad. There is a lot of shortages as well as there is a lot of room for improvement with regards to the caregivers itself. Due to the over population at the hospitals and the stress of the job itself, working under pressure, short staff…what happens is that they give care that is sub optimal I would say because they’re dealing with a bulk of people. They can’t give the quality care that you get in a private hospital”. (‘Low emerging middle class’ group) “Sometimes you find the nurses shouting but you don’t know what’s the problem”. (‘Poor’ group) “But some of them shouting at the people who are taking their children to the clinic, some children are missing immunization they didn’t get it, ja (yes) they used to shout” (‘Poor’ group)	“I would like to see the doctor explain more about the medicine he’s giving you and the side effects. I don’t like reading pamphlets. I like to know from them. Because very few of them explain what the ingredients are or ask you, are you allergic to it”. (‘Realised middle class’ group) “You going to top specialist, it’s the best, but you paying”. (‘Emerging affluent’ group) “I’ve always had good service from them. Never really had a problem with time or appointments or getting treatment on time and things… and off course in pharmacies”. (‘Realised middle class’ group) “I normally wait…It’s just that if I go for the emergency, maybe like for my kids, I normally wait at the hospital, at the Netcare”. (‘Realised middle class’ group) “Okay right now it’s a private clinic and I must say I was very disappointed, it was bad experience that I had in Private. I had to change doctors as a result, my kid (child) was misdiagnosed and I was thinking, this is a specialist doctor who should know what she should be doing and we ended up being hospitalised. They were carrying out different expensive procedures for a simple problem and eventually the person who ended up solving that problem came from a public clinic. So I was very disappointed in that…what I think private health care does is, they offer you the most expensive option and they don’t take time to really investigate the condition because each child is different. It’s just a ticking, ticking thing, you walk in, yes it’s a numbers thing, you find you have 20 of you waiting for the doctor and you don’t get that personal connection”. (‘Realised middle class’ group)

## Discussion

### Main findings

The study showed that all people have access to medicines and healthcare services irrespective their race or socio-economic status. Accessing healthcare facilities was not an issue as these were available in both urban and peri-urban areas. The extended travelling time for those in the townships indicated transportation was a barrier to accessing healthcare quickly. This may be attributed to these participants’ socio-economic status where they were reliant upon public transport.

The results from the focus group discussions showed that parents are naturally concerned when a child falls ill and are willing to spend out-of-pocket if needed to ensure the well-being of the child. This is in relation to both healthcare services and the acquisition of medicines. The study also showed that although many individuals in the ‘Poor’ group were accessing healthcare for their children through the public sector, many were acquiring medicines from a combination of public (clinics and hospitals) and private medicine outlets (dispensing doctors, hospitals, private pharmacies including retail supermarkets). Availability of medicines was problematic in the public sector and the general consensus was that most often participants were asked to purchase medicines from the private sector due to shortages or non-availability at the clinics. The ‘Poor’ group felt common, necessary medicines such as multivitamins and cough syrups were expensive, whilst other socio-economic groups found these affordable. Public sector clinics were most often used for immunizations because the service was offered free of charge.

It was found that the quality of medicines provided in the public sector was questionable as many participants mentioned medicines were diluted with water, bottles were opened when supplied and medicine containers did not have expiry dates on them. Concern with the quality of medicines led to distrust in the public sector and participants mentioned they preferred the medicines purchased from the private sector pharmacies over those provided in the public sector clinics. A possible measure to dispel this distrust is for prescribers to ensure they are consistent on prescriptions ensuring only the International Non-proprietary name of a medicine is entered. This is a practice employed in Scotland which has also reported an increase in prescribing efficiency for proton pump inhibitors, statins and renin-angiotensin inhibitor drugs [27]. The quality of medicines and services in the private sector was perceived as ‘good’ although these services were noted to sometimes be expensive. Some participants felt they did not receive adequate time and explanations from the doctors despite paying high prices for their services and wanted more meaningful relationships and some related their negative experiences.

The findings from these Focus group discussions are important for policy makers who can use this opportunity and information generated to understand patient perceptions and requirements. There is always room for improvement and betterment of healthcare and these discussions show that patients value their relationships with their healthcare providers in both sectors and are not currently getting this. Patients’ perceptions are vital to understanding how implemented medicines and healthcare policies are being received. The successes and/or failures of SA’s national medicines and essential medicines policies and the efficient functioning of the country’s healthcare system can be measured and improved upon in this way.

### Comparisons with other studies

A study conducted by Patel et al., 2012 [28] on the quality of generic medicines in SA found that participants described the quality of generics in terms of the effect of medicines which is the same as in this current study. Participants also used similar terms to describe quality such as “drug works” and these participants also acknowledged their limitations in assessing quality of medicines. Similarly Panado® was also a popular choice in brand. The study also revealed that there were concerns with quality of medicines but these reports were from the pharmacists working at a public sector medicine depot. The pharmacists felt that medicines intended for use at primary care facilities were not monitored as effectively as more expensive medicines and felt their complaints were not adequately addressed by the provincial procurement agency nor the Medicine Control Council (regulatory body for medicines and medicines registration in SA). The concluding statement of the study was that it was time to understand and address the problems with medicines within the social paradigm and for trust to be built with the agencies responsible for regulation of quality and safety of medicines [28].

Another study by Patel et al., 2009 on SA consumer perceptions of drug quality showed that generic medicines and medicines supplied without a fee from the state were viewed to be of poor quality and treated with suspicion. In addition, participants also used product attributes such as time and duration of effect on their symptoms to evaluate quality of medicines, similar to our study [29].

Patients do not use medicines in isolation. A range of factors such as social, cultural, political and economic issues influences their use, perceptions and opinions of medicines. It is for these reasons policies cannot be developed and managed in a vacuum and patients’ opinions, views and interests must form part of the policy decision-making processes for improved outcomes with quality and safety of healthcare [29]. SA can learn much in this respect from the examples discussed below.

The WHO process for essential medicine list decisions makes provision for patient advocacy groups to comment on numerous applications and draft recommendations although these are not used in the decision making part of the process. This is not currently considered in the SA Essential Medicines List decision making process and was a recommendation to the SA National essential medicines list committee by Perumal-Pillay and Suleman in their study investigating the selection of essential medicines in SA [30].

Much can also be learnt from the Consumer Focus Collaboration in Australia which was established in 1997. The intention of this collaboration was to foster an active partnership between consumers of healthcare and healthcare providers at a national level to strengthen the focus of consumers in National Health Service planning, delivery, monitoring and evaluation. The collaboration proposes that active consumer participation in decision making results in improved outcomes; that access to quality information enables decision making and promotes involvement of consumers in managing their own health; that active consumer participation results in more accessible and effective services; that effective consumer participation in quality improvement and service development tasks is obtainable through a range of methods; that effective participation uses methods that encourages participation by those individuals usually marginalised by mainstream health services; and that active involvement by all consumers at all levels of the development, implementation and evaluation of health strategies and programmes is vital to its success [31].

### The importance of patients’ perceptions

Patients’ opinions are useful for quality control mechanisms and quality improvement of services rendered by a healthcare system. Health care organisations must be responsive to those aspects of service most valued by patients. These include: access to healthcare and medicines; meaningful relationships with healthcare professionals, the provision of understandable information; and participation in the healthcare and treatment decisions taken for themselves and their children. An aspect of healthcare quality that has been recognised for its importance is the influence of patient perception. Although a patient’s perception of quality is often focused on the service aspects of healthcare, it correlates with objective measures of healthcare quality. The ability of a healthcare organisation to meet the demands of a patient in terms of convenience and provision of information and medicines has significant outcomes on the quality of healthcare it ultimately is able to provide [32].

### Strengths and limitations:

We were able to gather perspectives and opinions from 41 parents and/or guardians of children (representing 78 children) in the eThekwini region, on their observations and experiences with availability of medicines and healthcare at the various healthcare institutions they used including both public and private sectors of healthcare. The study sample only included urban and peri-urban areas in the eThekwini Municipality region of KwaZulu-Natal province. The sample did not include deep rural areas and valuable comments from these individuals have not been included. This was not possible due to the financial and time constraints for the study. It is therefore recommended that a follow-up study be conducted to include opinions from communities in these areas.

### What this study adds and recommendations

This research has highlighted parent and/or guardian perceptions on availability, affordability and quality of medicines and healthcare for children in the eThekwini region in SA. Although there have been previous studies on the quality of medicines in SA, this is the first research study in SA to specifically publish qualitative information on medicines for children from the parents’/guardians’ perspectives. Although the study sample in this research is limited to a specific region in SA, it provides a basis for further research to be conducted country-wide. This will allow for patient input into pharmaceutical and healthcare policy governing access to and availability of essential medicines and services within the country.

#### Recommendations for policy makers

What is severely lacking and what is desperately needed in South Africa is overall quality management in the country’s healthcare system, including both public and private sectors. This is evident from research conducted on medicines selection policies in both the public and private sectors of healthcare in SA described in both published and unpublished work by Perumal-Pillay and Suleman [30]. Their research shows that monitoring and evaluation of essential medicines policies by SA government organisations is very poor with no patient’s outcomes evaluations or opinions included in policy decision-making. There is also minimal monitoring and evaluation of medicines selection policies and impact on patient outcomes performed by private medical insurance companies in the private sector (unpublished work). In both these instances there was no provision made for the inclusion of patients’ opinions and perceptions in such policy development. A clear indication of the success of policy implementation is obtainable from those most affected by or using the policy. Since patients are the end users of the healthcare system, the inclusion and consideration of their opinions and perceptions in management strategies including pharmaceutical and healthcare policy development and evaluations can be very valuable in achieving positive outcomes both in terms of satisfaction with service delivery and with patients’ health outcomes. Policy makers must assume a strategic overview of quality within the context of the health system and focus on inclusion of patient opinions and requirements but within limits so as not to ultimately compromise quality of healthcare delivery.

To adequately assess access to medicines for children in SA, a quantitative study on availability, affordability and use of medicines for children needs to be conducted. The World Health Organization/Health Action International methodology for measuring medicines prices, availability and affordability can be used to achieve this. In the absence of funding for such a study, it is further recommended that SA strive to obtain generics at the lowest possible price during the tender process and also look into the Novartis Access programme, if needed, to increase affordability and hence availability of medicines, especially those for children, to enhance universal access. Further studies are therefore required to fill this gap.

## Conclusions

A balanced approach in pharmaceutical and healthcare policy making should include public opinion. The public can only participate in such processes if their voices are heard. This can be achieved by incorporating perceptions from users of healthcare services. There is great value in public participation in policy decision making as it provides the public with a sense of trust, not being left out and accountability. It has the potential to positively impact on patient behaviours, rational use of medicines and services and overall promotion of health. This study captures this qualitative aspect of gathering patient perceptions for policy recommendations and calls for a quantitative study to be conducted to provide a full picture of access to medicines for children in SA.

## Additional file

Additional file 1: Discussion guide for Focus group discussions with Parents/legal guardians. (DOCX 19 kb)

## Abbreviations

SA: South Africa
WHO: World health organization

## Footnotes

1 At the time of the study R13 = 1 US$
