# Supplementary material for: Parents’ and guardians’ perceptions on availability and pricing of medicines and healthcare for children in eThekwini, South Africa – a qualitative study
Source: BMC Health Serv Res. 2017 Jun 19;17:417. doi: 10.1186/s12913-017-2385-y (PMC5477259; doi:10.1186/s12913-017-2385-y)
Supplement: Additional file 1: — Discussion guide for Focus group discussions with Parents/legal guardians. (DOCX 19 kb) [file 12913_2017_2385_MOESM1_ESM.docx]

**Discussion guide for Focus group discussions with Parents/legal guardians^[[1]](#footnote-1)^**

| **FGD# Data Collection Sheet: Participant Demographic Information** | |
| --- | --- |
| Participant Name/Study ID: |  |
| Age: |  |
| Gender: | □ Male □ Female |
| Relationship to head of household: |  |
| Level of education: | □ No formal education □ Primary □ Secondary □ Tertiary (University/Technicon) |
| Occupation: |  |
| Level of Income (per household per month): | □ R0 – R4533 □ R4534 – R12 644 □ R12 645 – R30 328 □ R30 329 – R52 593  □ R52 594 – R71 992 □ R71 993 – R1 108 20 □ > R1 108 21 |
| Number and ages of children in the household: | Number =  Ages = |
| Marital Status of each of the household members who live in the house: |  |

1. Do you have private medical insurance/medical aid?
2. Do you visit a public or private health care facility?
3. Do you visit a health care facility for acute illnesses and/or chronic illnesses for your children?
4. Do you obtain medicines from your health care facility and/or a private pharmacy?
5. How much does your household pay for medicines used to treat these illnesses annually?
6. Were all the prescribed medicines for these illnesses available at the health care facility you use or did you have to obtain some medicines elsewhere, such as a private pharmacy?
7. Was the full cost for the medicines covered by health insurance/medical aid or was there out of pocket expenditure/a co-payment you had to pay in? can you estimate the total annual out of pocket expenditure for the household.
8. How often do you purchase over the counter medicines for your children? For which conditions? And what is this total cost per annum/year?
9. Are your children immunized at a public or private institution? If immunized privately, are these costs covered by the medical insurance and in part/full?
10. What is your opinion on the availability of children’s medicines at the facility you visit?
11. What is your opinion on the affordability of the medicines prescribed/required?
12. What is your opinion on the quality of health care your child receives?
13. What is your opinion on the quality of the medicines you purchase?
14. Travel time to health care facility (<15mins; >1 hour). Is this a public or private facility?

1. Adapted from the standard WHO access to and use of medicines survey employed in the following study: Republic of Uganda: Access to and use of medicines in Uganda (2008) http://apps.who.int/medicinedocs/documents/s16374e/s16374e.pdf (2008). Accessed 03 February 2013. [↑](#footnote-ref-1)
